# Post-Pandemic Surges in Public Trust in the United Kingdom

**DOI:** 10.3390/bs15091193

**Published:** 2025-09-01

**Authors:** John Rose, Jason Reid, Lisa Morton, Sasha Stomberg-Firestein, Lisa Miller

**Affiliations:** Spirituality Mind Body Institute & Clinical Psychology Program, Teachers College, Columbia University, New York, NY 10027, USA; jdr2182@tc.columbia.edu (J.R.); em3756@tc.columbia.edu (L.M.); sss2181@tc.columbia.edu (S.S.-F.); lfm14@tc.columbia.edu (L.M.)

**Keywords:** public trust, civic institutions, artificial intelligence, healthcare, global trait, COVID-19 pandemic

## Abstract

Trust in public institutions was challenged during the COVID-19 global pandemic, with widespread mistrust towards healthcare institutions as well as fellow public institutions. Concurrently, a new public institution or social tool, mass-market artificial intelligence (AI), more broadly emerged, which too may be a target of fluctuating public trust. Using national survey data from the United Kingdom’s Centre for Data Ethics and Innovation (survey year: 2022, N = 4320; survey year: 2023, N = 4232), we explore the level of trust in civic institutions (healthcare, non-healthcare, and AI) during and immediately after the COVID-19 pandemic in the United Kingdom using a naturalistic quasi-experimental design. At both waves (2022 and 2023), principal component analysis and structural equation modeling over thirteen public institutions and AI variables confirmed three factors (or domains) of public trust: trust in healthcare institutions, trust in fellow civic institutions other than healthcare, and trust in AI. Measurement invariance testing of mean levels of public trust along each distinct component revealed that as compared with 2022, in 2023, (1) trust in healthcare institutions and in fellow civic institutions other than healthcare significantly increased and (2) trust in AI remained approximately level. Next, latent profile modeling revealed four levels of a common public trust profile, with all three domains of public trust being normatively closely associated. Taken together, these results suggest that a psychological stance of public trust, PT, may increase after a societal crisis.

## 1. Introduction

### 1.1. Background and Rationale

A large body of research has demonstrated the importance of public trust in fostering a reasonably socially cohesive, functional, and flourishing society (Catterberg, 2006; Hudson, 2006; Mishler & Rose, 2001; Olagoke et al., 2020; Rotter, 1980; Spadaro et al., 2020). However, limited research has been conducted on the nature of public trust. Generally speaking, might public trust substantially tend to differ by civic institution, or might public trust be a global human quality or characteristic, much like intelligence (Spearman, 1961) or character (Barton & Miller, 2015; Ford et al., 2023)?

If indeed public trust may be a common psychological stance, then how, under times of cultural stress or challenge to specific civic institutions, might those specific institutions or fellow institutions be contained in public trust? Following times of societal strain, does public trust increase?

If indeed trust is an overarching stance, then might we anticipate that public trust surges when stress is lifted? Past research has indicated that institutional trust may fluctuate with crisis and can increase post-stress depending on institutional communication and the course of the crisis (Coombs, 2007), with some theorists seeing crisis as an opportunity to increase trust (Ulmer et al., 2015). Here, we adapt the naturalistic societal stressor to public trust of the global COVID-19 pandemic upon a range of major public institutions in the United Kingdom (UK), made possible through the generous sharing of data by the UK Center for Data Ethics and Innovation.

#### 1.1.1. Construct of Trust

Trust has been examined from multiple perspectives, including developmental psychology, interpersonal psychology, personality theory, and political science. Erik Erikson’s first stage of development proposes that infants have either responsive or unresponsive caregivers and thus develop a sense of trust or mistrust, respectively (Erikson, 1993). Attachment theory proposes that attachment style, which develops in infancy through caregiver interactions, also dictates how individuals trust (Ainsworth & Bell, 1970). Trust has been determined to be a precursor of honesty and transparency (Mellinger, 1956). Individuals high in trust are happier, more well-adjusted, and less likely to steal and lie (Rotter, 1980). Trust development is fundamental to sound interpersonal functioning.

Researchers have also suggested that trust is primarily a personality trait that we learn from a young age, meaning that we might trust different objects in the same way (Freitag & Bauer, 2016; Uslaner, 2002). In fact, trust is one of the facets of agreeableness in the Big Five Inventory-2 (BFI-2; Soto & John, 2017).

#### 1.1.2. Public Trust

Public trust is essential to the functioning of democratic societies. This study has adapted Mishler and Rose’s (2001) definition of public trust as trust towards public institutions to act in the people’s best interests to create a functioning society. This definition is supported by research suggesting that trust supports social functioning through two mechanisms. First, both interpersonal and institutional trust are essential to social capital, which in turn promotes belongingness and social engagement (Newton, 2001; Putnam, 2001). Secondly, institutional trust promotes individuals internalizing social norms and laws, fostering civic engagement and relational functioning (Levi & Stoker, 2000; Tyler, 1990). Furthermore, research suggests that public trust and well-being are linked (Catterberg, 2006). Public trust is also linked to institutional performance: trust in political institutions begets adherence to rules set by them, which is associated with more effective operation (Levi & Stoker, 2000). Conversely, when individuals suspect corruption in the government, their public trust is decreased (Catterberg, 2006).

To distinguish trust in AI from traditional objects of public trust (the government, healthcare institutions, private and public organizations serving the public interest, etc.), this study will refer to these traditional objects of public trust as trust in civic institutions. Public trust, therefore, will be considered the umbrella term comprising trust in civic institutions and trust in AI. This conceptualization is supported by research that suggests that not only is trust in AI built on the foundation of institutional trust, but also public trust contains both traditional civic institutions and AI (Bullock et al., 2025; Knowles & Richards, 2021).

#### 1.1.3. Trust in Civic Institutions

Trust in civic institutions encompasses the extent to which individuals trust institutions in the public sector to act in their best interests (Bosio, 2023). This construct includes a similar concept: institutional trust, which is simply how individuals trust the government to create conditions conducive to a successful life (McKnight & Chervany, 2000). A cultural element is involved in trust—how and to what extent individuals are shaped by their culture’s norms and values (Thanetsunthorn & Wuthisatian, 2019).

Trust in civic institutions is well known to have a variety of benefits for societies—it is intimately intertwined with interpersonal trust and well-being, which are higher in societies with high public trust because it provides a feeling of security (Spadaro et al., 2020). Trust in civic institutions has been shown to be crucial to individuals’ mental and physical health and overall well-being, in part because it is associated with increases in individuals’ perceived self-efficacy (Hudson, 2006; Olagoke et al., 2020).

During the COVID-19 pandemic, trust in civic institutions was a core moderator of pandemic distress: those high in public trust showed lower levels of mental illness (Olagoke et al., 2020). Research from the United Kingdom (UK) showed that distrust in the government and the healthcare system was widespread in the UK during the pandemic (Enria et al., 2021; Fancourt et al., 2020). Although trust in civic institutions rose initially during the primary stages of the lockdown in April 2020, it declined the rest of the year (Davies et al., 2021). While pandemic restrictions had begun to be lifted by the beginning of 2022 (The Week, 2022), trust in civic institutions was likely still suffering, especially with Boris Johnson’s indictment in the public sphere (Kirka & Hui, 2023).

In 2022 and 2023, the UK made several efforts to engage with widespread public distrust. The UK government introduced a Resilience Framework in December 2022 to strengthen government systems in the face of civil emergencies, resulting in a yearly statement to parliament beginning in 2023 (HM Government, 2022). The UK COVID-19 Public Inquiry was also launched in 2022, seeking to better ascertain the government’s response to the pandemic and its impact on the UK to increase preparedness for future crises. Public hearings began in 2023 on resilience and preparedness (Hallett, 2024). These efforts, while significant, represented an attempt to recognize public distrust of institutions but did not focus on directly improving public trust.

A surge of research on trust in civic institutions and pandemic-related attitudes and behaviors using latent profile analysis was produced during the COVID-19 pandemic, finding that public trust was associated with closely following pandemic-related protocols (Kleitman et al., 2021; Verboord, 2024). This research provided more evidence that trust in government significantly declined during the pandemic (Reid et al., 2024). The harm the pandemic caused to institutional trust was a global, cross-cultural phenomenon: countries across the world experienced declines in public trust despite differentiated responses by country (Devine et al., 2021; Saaranen, 2024). The COVID-19 pandemic, therefore, represents a specific case of stress on trust in civic institutions.

Past analyses examining trust in civic institutions have frequently been sector-specific: they focus on public trust in government, healthcare, or the media, finding that trust in these areas has a strong cultural element and is influenced by current events and government actions (Davies et al., 2021; Kaasa & Andriani, 2022; Liu & Nusslock, 2018).

While this sector-specific research is crucial to understanding various facets of society, general public trust must also be examined because trust in civic institutions and civic engagement contribute to societal success (Marozzi, 2015).

#### 1.1.4. Trust in AI

Contemporaneous with pandemic-induced public distrust, the AI boom accelerated. During this time, generative AI, a type of AI that can produce content, grew exponentially in the form of large language models and became more available to the public (Griffith & Metz, 2023). Despite this rapid innovation and the subsequent excitement, generative AI does come with risks: scientists are concerned about misinformation, safety, and misuse of these systems (Stokel-Walker & Van Noorden, 2023). Furthermore, a 2019 study investigating trust in AI across the European Union found that concerns about the safety of AI systems negatively affected trust in AI (Omrani et al., 2022).

Given the idea that trust affects adherence and engagement with an institution (Pagliaro et al., 2021), we might infer that how individuals trust AI to serve their interests impacts how they interact with and adopt various AI-based systems (Lee & See, 2004). While there is not a plethora of research on trust in AI due to its relative novelty, researchers have recently created and validated the AI Attitude Scale (AIAS-4) to measure individuals’ general perception of AI (Grassini, 2023). It has also been found that the extent to which an individual trusts AI is strongly related to their personality and is dependent upon the field in which AI is being implemented (Aoki, 2020; Riedl, 2022).

While trust in AI has begun to be studied at length, research has not yet addressed how AI is conceptualized. So, the question arises, is AI perceived as a civic institution like health care, business, or government? As AI becomes more widely used by the public and more integrated into society and public interest work, it may be regarded as a public good and thus an object of public trust (Züger & Asghari, 2023). Furthermore, research has suggested that AI should be viewed as a unique social and cultural institution due to its ability to coordinate decision-making and information gathering (Farrell et al., 2025).

The field of trust in AI, and more broadly how individuals consider AI, is budding, but more empirical research is needed to better understand this phenomenon as AI is rapidly expanding.

### 1.2. Objectives

Other studies have investigated the relationship between trust in civic institutions and trust in AI. Y.-N. K. Chen and Wen (2021) found that trust in AI is significantly correlated with and enabled by trust in civic institutions and attitudes towards the government. Montag et al. (2023), on the other hand, found that trust in AI and trust in fellow humans (a component of public trust as civic institutions are run by humans) are separate constructs as they stem from different neural pathways. More empirical research is necessary to determine the full relationship between public trust and trust in AI. Specifically, we ask the following questions:

(1). Might healthcare institutions, fellow civic institutions (other than healthcare), and AI be perceived as distinct dimensions of public trust among those in the UK and, in turn, show distinct levels of trust during the pandemic?

(2). Post-pandemic, might the level of trust among those in the UK in each dimension increase, decline, or remain constant as compared to mid-pandemic?

(3). Is there evidence for the components of public trust going hand-in-hand, suggesting an overall psychological stance of public trust among those in the UK?

This present study provides an important and timely inquiry into the nature of public trust in the UK mid-pandemic and post-pandemic, encompassing both civic institutions and AI.

## 2. Methods

### 2.1. Study Design

This study uses data from the 2022 and 2023 collections of the Public Attitudes to Data and AI (PADAI) Tracker Survey. The PADAI Survey was conducted by Savanta on behalf of the UK’s Centre for Data Ethics and Innovation (CDEI). Data was collected via an online, self-report survey of questions about attitudes towards and trust in AI, data, and civic institutions. The 2022 and 2023 collections were the second and third waves of the collection. The first wave, collected in 2021, contains similar but not matching questions, so it could not be included in this analysis. Ethics approval was not required under the EU General Data Protection Regulation and the 2018 UK Data Protection Act, as no personal data were collected and this research was considered low risk (European Union, 2016; Legislation.gov.uk, 2018). The PADAI Tracker Survey (waves 2 and 3) followed standards ensuring voluntary participation, anonymity, informed consent, and data protection.

### 2.2. Setting

The wave two survey was conducted from 27 June to 18 July 2022, and the wave three survey was conducted from 11 to 23 June 2023. Participants were recruited through an existing pool of survey participants, supplied by Cint. The pool comprised pre-screened individuals who chose to take part in online surveys and research. Participants were compensated for their involvement in this study via PayPal or Amazon vouchers. Different participants were surveyed in each wave, so a purely longitudinal analysis could not be conducted.

### 2.3. Participants

Participants comprised a sample of UK adults over the age of 18. Quotas were used in sampling to ensure the sample was representative of those who would take part in an online survey in the UK according to age, gender, socio-economic status, ethnicity, and region.

### 2.4. Variables

We selected questions present in both the 2022 and 2023 datasets to analyze trust in civic institutions and trust in AI over time. The following question was used to measure trust in civic institutions: “To what extent, if at all, do you generally trust the following organisations to act in your best interest?” Participants were asked this question regarding the following organizations: “The NHS”, “The Government”, “Academic researchers at universities”, “Social media companies (e.g., Facebook, Instagram, TikTok, Twitter)”, “Big technology companies (e.g., Amazon, Microsoft, Google, Apple)”, “Utilities providers (e.g., gas, electricity, broadband)”, “Regulators (e.g., the Financial Conduct Authority, Ofsted, Ofcom)”, and “Researchers at pharmaceutical companies”.

The following questions were used to measure trust in AI: “Based on your current knowledge and understanding, what impact do you think Artificial Intelligence (AI) will have overall on society?” and “To what extent do you think the use of Artificial Intelligence (AI) will have a positive or negative impact for the following?”: “How fairly people are treated in society”, “How easy it is to do day-to-day tasks (e.g., plan travel routes, order food, find information online)”, “Job opportunities for people like you and your family”, and “Healthcare for people like you and your family”.

### 2.5. Data Sources and Measurement

Data was collected via an online, self-report survey of questions about attitudes towards and trust in AI, data, and civic institutions administered in 2022 and 2023. Participants were recruited through an existing pool of survey participants, supplied by Cint. The pool comprised pre-screened individuals who chose to take part in online surveys and research.

Participants endorsed their trust in each civic institution on a 5-point Likert scale where 1 = “Do not trust at all” and 5 = “Trust a lot”. While these questions do not comprise a validated scale, they help gain insight into how people in the UK trust a range of civic institutions.

The first question on AI, the overall impact of AI on society, was asked to participants on a 10-point Likert scale where 1 = “AI will have a very negative impact on society” and 10 = “AI will have a very positive impact on society”. The latter four questions, the impact of AI on fair treatment, ease of day-to-day tasks, job opportunities, and healthcare, were asked on a 5-point Likert scale where 1 = “Very negative” and 5 = “Very positive”. While these questions do not comprise a validated scale, they help gain insight into how people in the UK trust various aspects of AI.

### 2.6. Bias

Efforts were undertaken to address bias throughout the research process. Selection bias was minimized by using a demographically representative sample of UK individuals willing to participate in an online survey while ensuring eligibility. Information bias was addressed by using consistent survey formatting. Response bias was addressed through using neutral language and ensuring anonymity by not collecting personal data.

### 2.7. Study Size

We were appreciative to benefit from a large dataset (n > 4000) each year from our partnership with the UK government.

### 2.8. Quantitative Variables

All quantitative variables were handled as continuous in our analyses. Variables were examined for outliers. Higher levels of each variable were considered as higher levels of each construct (e.g., a score closer to 5 on the trust in government item was treated as a higher level of trust in government).

### 2.9. Statistical Methods

#### 2.9.1. Principal Component Analysis

We conducted a principal component analysis (PCA) with Varimax rotation using SPSS version 29.0 (IBM Corp., 2022) on both the 2022 and 2023 data to determine if the survey’s questions about trust in civic institutions and trust in AI reduced to several core components. PCA is one of the oldest and most widely used methods in exploratory factor analysis (Jolliffe & Cadima, 2016). PCA condenses large datasets to several core variables that explain the greatest amount of variance in the dataset. In essence, PCA allows researchers to simplify large datasets by identifying the most important underlying variables in the dataset. These variables, known as principal components, are linear combinations of the original variables (Abdi & Williams, 2010). Varimax rotation is the most common and widely accepted type of orthogonal rotation that maximizes loadings and thus allows us to clearly determine which variables load onto which components (Howard, 2016).

In deciding on the number of components to retain, we utilized several methods. We conducted a thorough visual inspection of the scree plot. We also examined the eigenvalues of each component and closely examined those components with eigenvalues above one, in line with the recommendation of Kaiser (1960). We sought to determine the most parsimonious yet comprehensive solution.

#### 2.9.2. Structural Equation Modeling and Measurement Invariance Testing

We conducted a structural equation model (SEM) across the 2022 and 2023 data, using year (2022 and 2023) as the grouping variable. The model was specified using the three latent variables determined from the PCA: trust in AI, trust in civic institutions other than healthcare, and trust in healthcare institutions. The SEM was conducted using the *lavaan* package version 0.6-17 in R with robust maximum likelihood (MLR) (Rosseel, 2012). A multi-group Multiple Indicators Multiple Causes (MIMIC) model was used to control for the effects of age, ethnicity, and gender on the three latent factors by using regression analysis. Following approaches outlined in prior research (F. F. Chen, 2007; Cheung & Rensvold, 2002; Hu & Bentler, 1999), chi-square difference tests were conducted, but model fit was primarily evaluated using several commonly used indices, namely Comparative Fit Index (CFI), Root Mean Square Error of Approximation (RMSEA), and the Standard Root Means Square Residual (SRMR).

In accordance with good practice in SEM (Kline, 2010; Tabachnick & Fidell, 2013), the data was cleaned in order to identify and exclude extreme outliers due to their disproportionate effect on SEM with large samples such as this one. Prior to model estimation, all indicator variables were standardized. Those cases with extreme values of an absolute value z-score of more than seven were flagged as outliers and excluded from the SEM. The total number of cases flagged was 277.

Measurement invariance testing of the three-factor model across the two-wave dataset was subsequently conducted. Following the recommendations of Brown (2015) and Byrne et al. (1989), we conducted a stepwise approach in completing configural invariance, metric invariance, and scalar invariance testing. Partial scalar variance was established after freeing the intercepts of two variables to vary across groups (trust in the NHS and trust in government) due to evidence of non-invariance (change in CFI > 0.01; F. F. Chen, 2007). Establishing partial scalar invariance allows for the comparison of the latent means in 2022 and 2023.

#### 2.9.3. Latent Profile Analysis

A person-centered approach was used to determine how these components appeared in individuals. Latent profile analysis (LPA) is a person-centered, latent variable modeling method that seeks to identify clusters of individuals that have similar scores across a set of variables (Pastor et al., 2007). LPA identifies distinct profiles of trust in the 2022 and 2023 samples. These profiles would provide insight into how people might trust civic institutions and AI. We used the three components with loadings determined by the PCA as the variables because we are exploring these new, separated constructs, and thus it allows us to maintain flexibility in identifying profiles while handling complex item loadings. Using the PCA loadings rather than the CFA loadings allows the LPA to more accurately reflect the data and avoid any error from model misspecification in the latent variables from the CFA (Lubke & Muthén, 2005). The LPAs were conducted using *tidyLPA* version 1.1 (Rosenberg, 2021). *TidyLPA* allows researchers to perform the most commonly used LPAs in R. It does so by allowing researchers to manipulate and control for the variances and covariances of the profiles and variables. Pastor et al. (2007) can be consulted for a more in-depth exploration of LPA models.

*TidyLPA* version 1.1 allows for researchers to specify four models, each representing a different combination of parameters. Model A is the most restrictive, as variances are held equal across profiles and covariances between the variables are fixed at zero. Model B is less restrictive, with variances varying freely across profiles and covariances fixed at zero. Model C is even less restrictive, as variances are allowed to vary freely across profiles, and covariances of the three variables are estimated and held equal across profiles. Model D is the most flexible, with both variances and covariances varying freely within variables and between profiles.

Following the approaches recommended by Pastor et al. (2007), De Souza Marcovski and Miller (2023), and Ford et al. (2023), we examined the fit indices and model fit solutions from the four parameterization combinations to select a model. We analyzed the Akaike Information Criterion (AIC; Akaike, 1987), the Bayesian Information Criterion (BIC; Schwarz, 1978), entropy, and the bootstrapped likelihood ratio test (BLRT; Nylund et al., 2007) as the main indices. We sought to determine the final model by not only evaluating these metrics but also conducting thorough visual inspections of each model.

## 3. Results

### 3.1. Participants

A total of 4320 participants were surveyed in wave two (2022), and 4232 participants were surveyed in wave three (2023). Different participants were surveyed in each wave.

### 3.2. Descriptive Data

Participants reported their age, sex, ethnicity, occupation, and region in which they lived in both the 2022 and 2023 surveys. In 2022, the mean age was 48.73, with 10.1% of participants between the ages of 18 and 24, 16.3% between the ages of 25 and 34, 16.8% between the ages of 35 and 44, 17.0% between the ages of 45 and 54, 16.1% between the ages of 55 and 64, 14.9% between the ages of 65 and 74, and 8.9% over the age of 75. In terms of gender, 51.8% of participants identified as female, 47.6% as male, 0.4% identified in another way, and 0.2% did not disclose their gender. With regard to ethnicity, 80.5% identified as White English/Welsh/Scottish/Northern Irish/British/Irish, 0.1% identified as Gypsy or Irish traveler, 2.2% identified as any other White background, 3.5% as having multiple ethnicities, 6.3% as Asian, 3.6% as Black, 1.2% as another ethnicity, and 0.7% did not disclose their ethnicity. In terms of location, 2.5% of participants were from Northern Ireland, 9.0% were from Scotland, 5.1% were from Wales, 10.4% were from London, 18.8% were from Southern England, 25.5% were from the Midlands, and 28.7% were from the North. With regard to occupation, 4.8% of participants worked in a high managerial, administrative, or professional role (e.g., doctor, lawyer, medium/large company director); 17.7% worked in an intermediate managerial, administrative, or professional role (e.g., teacher, manager, accountant); 20.9% worked in a supervisor, administrative, or professional role (e.g., police officer, nurse, secretary, self-employed); 14.2% worked as a skilled manual worker (e.g., mechanic, plumber, electrician, lorry driver, train driver); 12.6% worked as a semi-skilled or unskilled manual worker (e.g., waiter, factory worker, receptionist, laborer); 2.9% were housewives or househusbands; 6.3% were unemployed; 1.7% were students; and 19.0% were retired.

In 2023, the mean age was 48.98, with 10.7% of participants between the ages of 18 and 24, 16.6% between the ages of 25 and 34, 15.8% between the ages of 35 and 44, 16.8% between the ages of 45 and 54, 15.8% between the ages of 55 and 64, 13.2% between the ages of 65 and 74, and 10.9% over the age of 75. In terms of gender, 53.8% of participants identified as female, 45.6% as male, 0.4% identified in another way, and 0.2% did not disclose their gender. With regard to ethnicity, 76.6% identified as White English/Welsh/Scottish/Northern Irish/British/Irish, 0.1% identified as Gypsy or Irish traveler, 2.3% identified as any other White background, 4.2% as having multiple ethnicities, 9.0% as Asian, 5.4% as Black African/British/Caribbean/other background, 1.5% as another ethnicity, and 0.9% did not disclose their ethnicity. In terms of location, 2.6% of participants were from Northern Ireland, 7.9% were from Scotland, 4.8% were from Wales, 13.5% were from London, 22.0% were from Southern England, 25.2% were from the Midlands, and 24.0% were from Northern England. With regard to occupation, 4.5% of participants worked in a high managerial, administrative, or professional role (e.g., doctor, lawyer, medium/large company director); 11.8% worked in an intermediate managerial, administrative, or professional role (e.g., teacher, manager, accountant); 22.0% worked in a supervisor, administrative, or professional role (e.g., police officer, nurse, secretary, self-employed); 17.0% worked as a skilled manual worker (e.g., mechanic, plumber, electrician, lorry driver, train driver); 14.0% worked as a semi-skilled or unskilled manual worker (e.g., waiter, factory worker, receptionist, laborer); 2.4% were housewives or househusbands; 7.5% were unemployed; 2.2% were students; and 18.7% were retired.

### 3.3. Outcome Data

Outcome data and analyses on primary variables are reported in Section 3.

### 3.4. Main Results

#### 3.4.1. Principal Component Analysis

**Figure 1:**
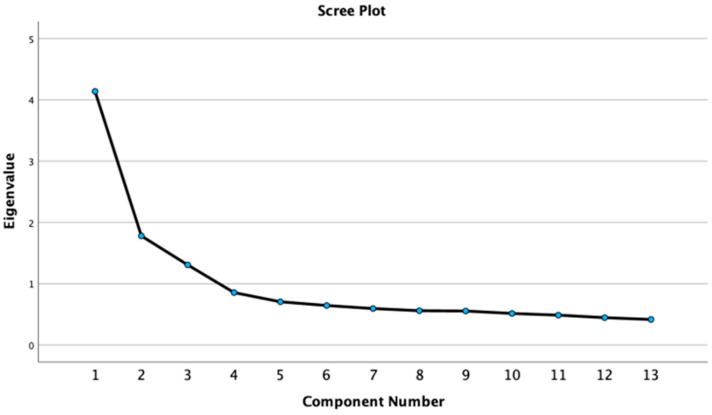
The 2022 PCA scree plot.

Our first objective was to see if the survey’s questions about trust in civic institutions and trust in AI reduced to several core components. Table 1 shows the results of the PCA for the 2022 data. The primary component with the highest eigenvalue of 4.137 accounted for 31.825% of the variance. The component with the second highest eigenvalue of 1.779 accounted for 13.685% of the variance. The component with the third highest eigenvalue of 1.307 accounted for 10.051% of the variance. In following Kaiser’s rule of keeping components with eigenvalues above one, we kept these three components. We proceeded to an examination of the scree plot in Figure 1 to confirm these findings. The scree plot exhibits a steep decline after component one, a decrease at a constant rate across components two, three, and four, and another slight drop-off after component four. However, because component four did not have an eigenvalue above one, we elected to keep only the first three components.

**Figure 2:**
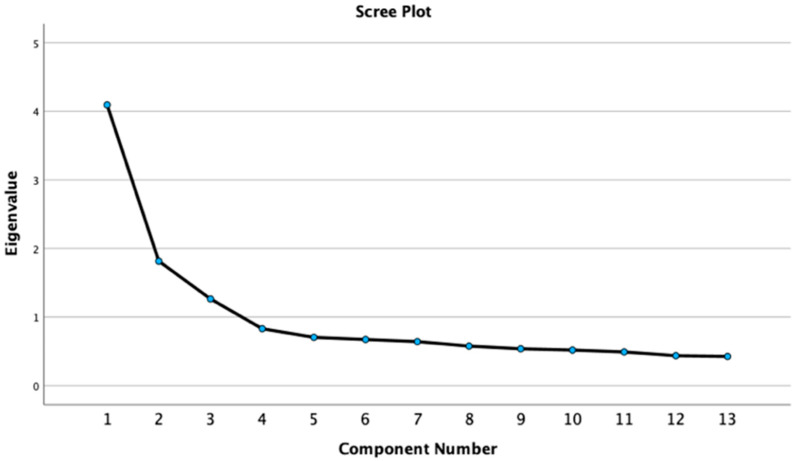
The 2023 PCA scree plot.

The number of components and the variance they accounted for stayed approximately consistent in the 2023 data. Table 2 shows the results of the PCA for the 2023 data. The primary component with the highest eigenvalue of 4.093 accounted for 31.483% of the variance. The component with the second highest eigenvalue of 1.814 accounted for 13.955% of the variance. The component with the third highest eigenvalue of 1.264 accounted for 9.723% of the variance. In following Kaiser’s rule of keeping components with eigenvalues above one (Kaiser, 1960), we kept these three components. We proceeded to an examination of the scree plot in Figure 2 to confirm these findings. The scree plot in Figure 2 exhibits a steep decline after component one, a decrease at a constant rate across components two, three, and four, and a slight drop-off after component four. However, because component four did not have an eigenvalue above one while the first three components did, we elected to keep only the first three components. The PCAs yielded remarkably similar results across 2022 and 2023, and thus our subsequent analyses of which components to retain were the same.

We proceeded to examine the component loadings to determine which survey questions loaded onto which components. Across 2022 and 2023, the loadings were consistent. Component one comprised the trust in AI questions: AI’s overall impact on society loaded 0.728 in 2022 and 0.748 in 2023. AI’s impact on how fairly people are treated in society loaded 0.748 in 2022 and 0.728 in 2023. AI’s impact on ease of day-to-day tasks loaded 0.735 in 2022 and 0.752 in 2023. AI’s impact on job opportunities loaded 0.721 in 2022 and 0.687 in 2023. AI’s impact on healthcare loaded 0.758 for 2022 and 0.768 for 2023.

Component two comprised trust in civic institutions other than healthcare. Trust in government loaded 0.632 in 2022 and 0.628 in 2023. Trust in social media companies loaded 0.724 in 2022 and 0.729 in 2023. Trust in big technology companies loaded 0.721 in 2022 and 0.661 in 2023, representing, while still minor, the largest change in loading between 2022 and 2023. Trust in utility providers loaded 0.690 in 2022 and 0.708 in 2023.

Component three encompassed trust in healthcare institutions. Trust in the NHS loaded 0.720 in 2022 and 0.670 in 2023. Trust in academic researchers at universities loaded 0.747 in 2022 and 0.743 in 2023. Trust in regulators loaded 0.546 in 2022 and 0.598 in 2023. Trust in researchers at pharmaceutical companies loaded 0.683 in 2022 and 0.624 in 2023. The complete component loadings can be found in Table 3.

The naturally occurring dimensions of public trust, therefore, were represented by the three dimensions of public trust: AI, civic institutions other than healthcare, and healthcare institutions.

#### 3.4.2. Structural Equation Modeling and Measurement Invariance Testing

Next, we sought to verify the three-factor model from the PCA using a MIMIC SEM while controlling. Despite a chi-square test indicating a misfit (χ^2^ (df = 304) = 2915.81, *p* < 0.05), there was an acceptable CFI (CFI = 0.867), and the RMSEA and SRMR indicate an acceptable fit (RMSEA = 0.051, RMSEA < 0.05 indicates an acceptable fit; SRMR = 0.041, SRMR < 0.5 indicates a good fit).

Factor loadings suggested the three-factor structure ascertained from the PCA was valid. Namely, component one was confirmed to be trust in AI. AI’s overall impact on society loaded 0.372 in 2022 and 0.415 in 2023. AI’s impact on how fairly people are treated in society loaded 0.723 in 2022 and 0.756 in 2023. AI’s impact on ease of day-to-day tasks loaded 0.787 in 2022 and 0.760 in 2023. AI’s impact on job opportunities loaded 0.721 in 2022 and 0.687 in 2023. AI’s impact on healthcare loaded 0.739 for 2022 and 0.732 for 2023.

Component two was confirmed to be trust in civic institutions other than healthcare. Trust in government loaded 0.479 in 2022 and 0.499 in 2023. Trust in social media companies loaded 0.591 in 2022 and 0.577 in 2023. Trust in big technology companies loaded 0.717 in 2022 and 0.698 in 2023. Trust in utility providers loaded 0.644 in 2022 and 0.639 in 2023.

Component three encompassed trust in healthcare institutions. Trust in the NHS loaded 0.620 in 2022 and 0.639 in 2023. Trust in academic researchers at universities loaded 0.023 in 2022 and 0.535 in 2023. Trust in regulators loaded 0.020 in 2022 and 0.598 in 2023. Trust in researchers at pharmaceutical companies loaded 0.036 in 2022 and 0.612 in 2023. It is worth noting that these latter three items loaded significantly lower in 2022 than in 2023, suggesting a difference in factor structure between 2022 and 2023 and indicating the differential in healthcare trust between 2022 and 2023. The complete component loadings can be found in Table 4.

Multi-group measurement invariance testing was subsequently conducted to test the differences in the latent variables between the 2022 wave and the 2023 wave. Partial scalar variance was established using the model laid out above. Measurement testing results are laid out in Table 5. Trust in AI did not significantly change from 2022 to 2023 (z = −0.393, *p* > 0.05). Trust in civic institutions other than healthcare significantly increased from 2022 to 2023 (z = 9.254, *p* < 0.01). Trust in healthcare institutions also significantly increased from 2022 to 2023 (z = 10.366, *p* < 0.01).

Overall, the substantial increase from 2022 to 2023 in the level of public trust in civic institutions and in healthcare institutions may suggest that public trust can rapidly increase following a time of public crisis. Specifically, coming out of the COVID-19 pandemic, trust in civic institutions and healthcare institutions greatly increased, pointing to the tendency for public trust to increase after stress.

On the other hand, the level of public trust in AI remained constant between 2022 and 2023.

Additionally, in adding gender, ethnicity, and age as covariates into the model, we were able to see the effect of these demographic variables on the latent variables in 2022 and 2023. Namely, the model showed that for trust in AI, older individuals reported a higher level (standardized β = 0.049, *p* < 0.05), women reported higher levels (standardized β = 0.071, *p* < 0.01), and there were no differences among ethnicities. The model showed that for trust in civic institutions other than healthcare, older individuals reported a lower level (standardized β = −0.117, *p* < 0.01), women reported a lower level than men (standardized β = 0.064, *p* < 0.01), and individuals identifying as mixed ethnicity (standardized β = 0.052, *p* < 0.01), Black (standardized β = 0.038, *p* < 0.05), and Asian (standardized β = 0.105, *p* < 0.01) had higher levels. The model showed that for trust in healthcare institutions, older individuals reported higher levels (standardized β = 0.090, *p* < 0.01), women reported a lower level than men (standardized β = −0.067, *p* < 0.01), and individuals identifying as Asian (standardized β = −0.101, *p* < 0.01), Black (standardized β = −0.101, *p* < 0.01), and another ethnicity (standardized β = −0.057, *p* < 0.01) had lower levels.

#### 3.4.3. Latent Profile Analysis

Our third goal was to examine profiles of individuals in the sample based off their trust in AI, trust in civic institutions other than healthcare, and trust in healthcare institutions. Table 6 displays results from the LPAs, including indices of model fit, including AIC, BIC, entropy, and BLRT. We coded the program to compute models with one through six solutions, which allowed for significant depth in the models while excluding models with more profiles, as they might detract from explanatory clarity. Lower values for AIC and BIC imply better model fit, while higher entropy values suggest greater accuracy of the model.

Our results show that model D (which allows varying variances and varying covariances), the least restrictive model, has the lowest AIC and BIC in both 2022 and 2023. However, due to its complex parameterization, the levels of entropy fall significantly below 0.7, meaning that individuals were not confidently placed in the correct profiles, so we discarded this model. Model C, the second least restrictive model, had quite low AIC and BIC but also had low entropy levels, so we discarded that model as well. For both models C and D, the models that had high enough levels of entropy had too few generated profiles (i.e., 2).

Model A, which kept equal variances and fixed covariances at zero, was the most restrictive model, not allowing the data to flow freely. As such, AIC and BIC were higher, but entropy levels were adequate, above 0.7. Model B, which allows variances to vary freely and covariances fixed at zero, had lower AIC and BIC than model A while also having entropy levels above 0.7. We closely examined the model B solutions in both 2022 and 2023. The five-class model was discarded because it provided more complexity without any individual insight as we sought the most parsimonious solutions, and the six-class model had too few individuals in the smallest class in both 2022 and 2023. The four-class solutions provided enough classes to gain a depth of understanding of the data (more so than the three-class model), relatively low AIC (22,460.82 in 2022 and 24,609.03) and BIC (22,624.93 in 2022 and 24,775.55 in 2023), sufficient entropy levels (0.76 in 2022 and 0.82 in 2023), BLRT less than 0.01 in 2022 and 2023, and approximately 5% or more of the sample in their smallest profile (12.4% in 2022 and 4.9% in 2023).

**Figure 3:**
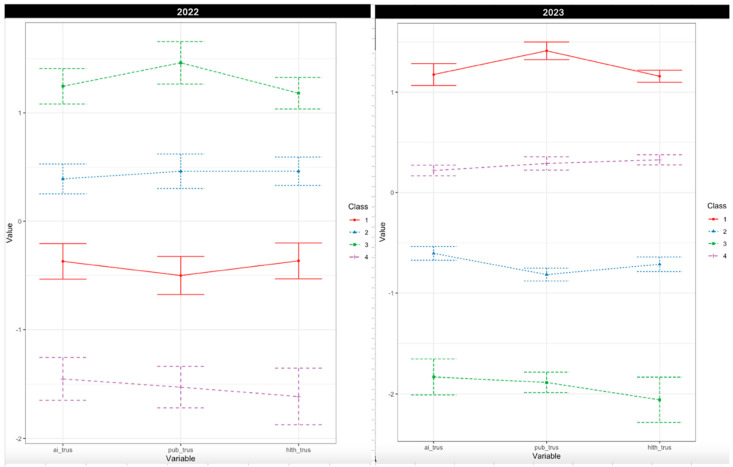
The LPA model B, four-class solutions from 2022 (**left**) and 2023 (**right**).

As seen in Figure 3, we identified (from top to bottom in the charts) the four classes as the high trusters (12.6% of the sample in 2022 and 47.5% in 2023), the medium–high trusters (39.7% of the sample in 2022 and 15.7% in 2023), the medium–low trusters (35.3% of the sample in 2022 and 32.0% in 2023), and the low trusters (12.4% of the sample in 2022 and 4.9% of the sample in 2023). While only 4.9% of the sample in 2023 were low trusters, the decline from 12.4% of the sample in 2022 is telling of the overall increase in trust in civic institutions and healthcare institutions from 2022 to 2023. We stopped our investigation at this point because the relationship between the profiles was clear.

Because the profiles are shown as parallel horizontal lines in the plotted model, as seen in Figure 3, we can conclude that trust is a global phenomenon: If one is high in one aspect of trust, they are more likely to be high in the other two aspects of trust as well. Similarly, if one is low in one aspect of trust, they are more likely to be low in the other two dimensions of trust. The PCA likely did not capture this global aspect of trust because it requires higher levels of correlation between items for them to load onto a single component. The LPA was able to capture the global nature of trust because it does not require correlations to be as high. Finally, the LPA findings suggest that global trust may vary in absolute level but does not vary by constitution or profile shape per se, as the slope across the four lines does not meaningfully differ.

## 4. Discussion

### 4.1. Key Results

The goals of the current study were to (1) determine whether objects of public trust are distinctly perceived and thus show distinct levels of trust during and after the pandemic, (2) assess whether the level of trust in each dimension increases, decreases, or remains constant after the pandemic as compared to during the pandemic, and (3) determine whether the levels of each dimension of public trust go hand-in-hand, suggesting an overall stance of public trust. We utilized a dimension-reduction technique in principal component analysis to determine the components of trust, a multi-group MIMIC SEM to confirm these factors and measurement invariance testing to compare the data across years, and a person-centered approach in latent profile analysis with the principal components as indicator variables to determine how these components appeared in participants.

The analyses showed that public trust during and immediately after the naturalistic stress of the COVID-19 pandemic comprised three separate factors: trust in AI, trust in civic institutions other than healthcare, and trust in healthcare institutions. Civic institutions other than healthcare and healthcare institutions were experienced similarly: both had higher means in 2023 than in 2022. Adding age, gender, and ethnicity as covariates allowed us to control for these demographic variables and examine differences in them.

The analyses also showed that individuals might not experience AI as the same type of human-driven civic institution. AI had yet a different slope, being flat and unchanged from 2022 to 2023, displaying that trust in AI did not change as trust in the human-driven civic institutions other than healthcare and trust in healthcare institutions did. This finding illustrates that AI might not be experienced as a human-run civic institution that undergoes fluctuations of trust in the face of stress (here, in the form of COVID-19). Trust in AI’s evolution also supports the idea that AI is a unique cultural and social institution (Farrell et al., 2025), as trust in AI does not evolve in the same manner as trust in civic institutions other than healthcare and trust in healthcare institutions.

Notably, trust in AI loads onto a different institution than trust in the institutions that create AI: trust in technology companies loads onto trust in civic institutions. This finding supports the idea that AI is a unique social and cultural institution experienced perhaps as non-human (Farrell et al., 2025).

The LPA suggests the existence in this sample of a common stance across public institutions. Individuals have the same levels of trust relative to the population mean in each dimension of public trust. The tendency to trust in one domain goes hand-in-hand with the tendency to trust in the others with respect to the population mean. The four-profile solution showed four parallel horizontal lines in the model, suggesting public trust differs in level but not in constitution or profile form. We named these profiles the high trusters, medium–high trusters, medium–low trusters, and low trusters. In effect, individuals appeared to trust AI, civic institutions other than healthcare, and healthcare institutions at the same relative level.

Trust in civic institutions other than healthcare and trust in healthcare institutions rapidly increased following COVID-19, displaying the nature of trust in human-run organizations to increase after a crisis.

### 4.2. Limitations

We clarify some study limitations. First, while this study draws on a naturalistic, quasi-experimental design (mid- and post-pandemic), there are no direct claims of causality when it comes to public health crises and trust in civic institutions. It was not possible to conduct a singular longitudinal latent profile analysis because the two waves of the UK survey were made up of separate individuals. Therefore, we could not make claims about how the specific profiles evolved from 2022 to 2023 but rather draw a general cohort inference. Future research might incorporate longitudinal latent profile analysis to determine how trust in AI and public trust evolve in individuals over time.

Due to the nature of the data, we were also unable to investigate the role of public evaluation of the actions of these institutions and thus were not able to make claims about them. We were also unable to investigate any other causal mechanisms of increasing trust, as the data was focused on general attitudes towards public institutions and AI. Nevertheless, we do not attempt to explain why the changes in public trust occurred, but we examine its structure (i.e., how public trust breaks down by type of institution) and its timing in connection with the end of the pandemic (i.e., public trust was higher in the year after the pandemic). However, we acknowledge that the UK government did make attempts to recognize public distrust (Hallett, 2024; HM Government, 2022), and it is certainly possible that the level of trust in 2022 and subsequently 2023 is higher than it may have been otherwise without the efforts of the UK government. Nevertheless, we cannot make any causal claims about how these efforts may have impacted the evolution of public trust. Future analyses may collect evaluations of specific governmental actions in order to conduct causal path analyses.

Additionally, the current study draws from data specifically collected in the UK of individuals willing to take part in an online survey in only two years, 2022 and 2023. Therefore, our findings about public trust are limited to the sample and those in the UK willing to take part in an online survey during this time period. As such, we were unable to examine public trust over the course of the entire pandemic. Future research should consider incorporating individuals from numerous, diverse countries and a range of cultures across numerous years to investigate the impact of culture and societal norms on trust over time.

As a note of clarification, the current study does not make claims on the absolute nor generalizable levels of trust (during this crisis of public trust perhaps as compared with other crises) because we did not use broadly validated measures of trust in AI or public trust. Future research should include validated measures such as the AI Attitude Scale (AIAS-4; Grassini, 2023) and the General Trust Scale (Yamagishi & Yamagishi, 1994). Furthermore, our inquiry is confined to trust in civic institutions and trust in AI due to the design of the original data collection. While we may extrapolate that a global construct of trust extends to other dimensions of trust, including interpersonal trust, we have yet to examine such claims. Future research should include validated measures of interpersonal trust, such as the Interpersonal Trust Scale (Rotter, 1967).

The larger data collection study did not assess knowledge about AI as a potential moderator for trust in AI. Given the rapidly developing and evolving nature of AI technology, there is a wide range in individuals’ understanding of what AI is and how it operates. Future studies may wish to examine this relationship as well as AI education as a potential means to bolster public trust in AI.

A variety of methods can be used for dimension reduction and confirmation. While we used principal component analysis followed by a multi-group MIMIC SEM, approaches such as principal axis factoring, exploratory factor analysis, or exploratory structural equation modeling may also be useful in determining and confirming the components of trust. Given that we sought a conceptually clear method with which to stratify the core questions as either loading or not loading on a component and maximize the variance accounted for, we elected to proceed with principal component analysis followed by multi-group MIMIC SEM. Future analyses may use a similar approach to McClintock et al. (2016) in utilizing an exploratory factor analysis followed by exploratory structural equation modeling, as this approach may provide a better fit and more differentiated factors. Furthermore, future research should consider a longitudinal approach to the study of public trust before, during, and after a societal crisis in order to ascertain public trust patterns in the face of crisis.

### 4.3. Interpretation

We suggest that an overarching psychological stance of trust exists in the sample, and several of its components are decreased under duress, specifically the COVID-19 pandemic, in 2022. In 2023, trust in AI, trust in civic institutions other than healthcare, and trust in healthcare institutions were at relatively higher levels and went hand-in-hand: high levels of one dimension of public trust are associated with high levels of the other dimensions.

In 2022, during a naturalistic time of greater pandemic public stress and institutional intervention, however, there were significantly lower levels as compared to 2023 in trust in healthcare institutions and trust in fellow civic institutions, while trust in AI was approximately the same. This finding of dimensions of different types of trust appearing at differentiated levels during the pandemic is supported by prior literature (Aassve et al., 2024). In 2022, we can still see the common psychological stance towards public trust, as these dimensions are at the same levels relative to the mean in the LPA, yet the individual dimensions of public trust are depressed.

Public trust, therefore, appears to be a common, global psychological quality, or characteristic in the sample, but more research may be necessary to establish it as a global trait similar to intelligence (Spearman, 1961) or character (Barton & Miller, 2015; Ford et al., 2023). We offer here the face-valid name of this construct to be public trust, PT.

What is most interesting about PT? Perhaps most striking is that human public trust increases and equilibrates across domains when stress is completed. An increase in trust post-crisis is well-documented across various settings and crises, including national security crises (Ojeda, 2016), public health crises (O’Malley et al., 2009), and natural disasters (Kotsila & Saravanan, 2017; Schilpzand, 2023). Our analyses exhibit lower levels of the dimensions of PT under stress, as opposed to post-stress, at which time trust in civic institutions other than healthcare and trust in healthcare institutions are significantly augmented. There is an increase to a higher level of trust, indicating that PT is fundamentally resilient.

### 4.4. Generalizability

This study investigates the different patterns of public trust driven by a naturalistic quasi-experimental design (mid- and post-pandemic), but we do not extensively explore why these patterns exist. Our findings of lower trust in civic institutions other than healthcare and trust in healthcare institutions in 2022 may be conceptualized in the context of prior findings that trust across Europe in political and healthcare institutions was significantly decreased in the middle of the pandemic as compared to the beginning because individuals perceived that civic institutions and healthcare institutions were ineffective in their efforts to mitigate the pandemic and restrictive measures were no longer necessary (Busemeyer, 2022; Weinberg, 2022). Furthermore, this research showed the connection between lower political trust and healthcare trust in the midst of the pandemic. However, since our research only examines public trust in 2022 and 2023, we cannot draw conclusions around this early pandemic research and our research that is focused on later pandemic and post-pandemic. Further research using data from throughout the pandemic is necessary to examine how public trust evolved throughout the entire pandemic.

This study is the first that we have encountered to suggest that public trust may be an overarching stance among a sample that surges when stress is lifted. However, more research across multiple countries and years is needed to determine if PT is indeed a global trait. We also cannot make determinations on whether this post-stress surge might have been different in the face of a different stressor.

This study also is the first we have encountered to examine the evolution of trust in civic institutions and trust in AI over time, side by side. Our findings are confirmatory of and complementary to those in Y.-N. K. Chen and Wen (2021)—we provided more support to the finding that trust in government (a component of trust in civic institutions) is correlated with trust in AI. Our findings also deliver insight into the idea that, although trust in humans and trust in AI may operate via different neurological pathways (Montag et al., 2023), as shown by the differential evolution of trust in AI as compared to trust in civic institutions other than healthcare and trust in healthcare institutions from 2022 to 2023, they are closely connected. We propose that a further exploration of the neurobiological mechanisms by which trust is securely established within a relational matrix during early developmental periods may yield further rich findings for how symbolic representations of trust are extended, more broadly, to AI and public institutions. Moreover, this line of inquiry might help uncover key aspects of resilience found in the observed effect of trust after periods of societal duress, such as the COVID-19 pandemic.

## 5. Conclusions

This current study explores the level of public trust across a range of civic institutions in the United Kingdom during the COVID-19 pandemic in 2022 and immediately after in 2023.

Overall findings suggest the existence of an overarching psychological stance of public trust, potentially a global PT, that increases and equilibrates when societal crisis is lifted. These findings suggest that public trust may thus be susceptible to trust-fostering interventions due to its movement. Researchers have suggested a variety of interventions for building public trust, including fostering communication with restructured institutional narratives and encouraging community-based responses (Correia, 2024; Hitlin, 2024). In times of stress, these findings suggest that there is hope yet for an increase in public trust.

## Figures and Tables

**Table 1:** Principal component analysis 2022 results.

	Initial Eigenvalues			Extraction Sums of Squared Loadings			Rotation Sums of Squared Loadings		
Component	Total	% of Variance	Cumulative %	Total	% of Variance	Cumulative %	Total	% of Variance	Cumulative %
1	4.137	31.825	31.825	4.137	31.825	31.825	2.832	21.788	21.788
2	1.779	13.685	45.510	1.779	13.685	45.510	2.317	17.820	39.608
3	1.307	10.051	55.561	1.307	10.051	55.561	2.074	15.954	55.562
4	0.855	6.578	62.139						
5	0.705	5.423	67.562						
6	0.644	4.956	72.518						
7	0.595	4.575	77.093						
8	0.559	4.303	81.396						
9	0.554	4.264	85.660						
10	0.514	3.958	89.618						
11	0.487	3.747	93.365						
12	0.446	3.431	96.796						
13	0.417	3.204	100.000						

**Table 2:** Principal component analysis 2023 results.

	Initial Eigenvalues			Extraction Sums of Squared Loadings			Rotation Sums of Squared Loadings		
Component	Total	% of Variance	Cumulative %	Total	% of Variance	Cumulative %	Total	% of Variance	Cumulative %
1	4.093	31.483	31.483	4.093	31.483	31.483	2.817	21.666	21.666
2	1.814	13.955	45.438	1.814	13.955	45.438	2.308	17.754	39.420
3	1.264	9.723	55.161	1.264	9.723	55.161	2.046	15.741	55.161
4	0.830	6.381	61.542						
5	0.704	5.412	66.954						
6	0.672	5.171	72.125						
7	0.641	4.933	77.058						
8	0.576	4.430	81.488						
9	0.537	4.128	85.616						
10	0.518	3.986	89.602						
11	0.491	3.773	93.375						
12	0.436	3.355	96.730						
13	0.425	3.270	100.000						

**Table 3:** Component loadings for the 2022 and 2023 principal component analyses.

	2022 Components			2023 Components		
	1	2	3	1	2	3
NHS trust	0.071	0.038	0.720	0.020	0.089	0.670
Government trust	0.089	0.632	0.149	0.150	0.628	0.170
Academics trust	0.125	0.081	0.747	0.133	0.030	0.743
Social media companies trust	0.152	0.724	−0.008	0.136	0.729	0.008
Big technology companies trust	0.132	0.721	0.229	0.103	0.661	0.295
Utility providers trust	0.121	0.690	0.187	0.115	0.708	0.228
Regulators trust	0.120	0.389	0.546	0.083	0.333	0.598
Pharmaceutical researchers trust	0.104	0.282	0.683	0.107	0.277	0.624
AI’s overall impact	0.728	0.193	0.089	0.748	0.197	0.088
AI’s impact on fair treatment	0.748	0.249	0.014	0.728	0.313	−0.018
AI’s impact on daily tasks	0.735	−0.029	0.229	0.752	−0.041	0.233
AI’s impact on job opportunities	0.721	0.240	−0.051	0.687	0.332	−0.121
AI’s impact on healthcare	0.758	0.001	0.246	0.768	−0.025	0.233

**Table 4:** Component loadings for the structural equation model.

Item	Latent Factor	2022 Loading	2023 Loading
AI’s overall impact	AI_TRUST	0.372	0.415
AI’s impact on fair treatment	AI_TRUST	0.723	0.756
AI’s impact on daily tasks	AI_TRUST	0.787	0.760
AI’s impact on job opportunities	AI_TRUST	0.693	0.692
AI’s impact on healthcare	AI_TRUST	0.739	0.732
Government trust	CIVIC_TRUST	0.479	0.499
Social media companies trust	CIVIC_TRUST	0.591	0.577
Big technology companies trust	CIVIC_TRUST	0.717	0.698
Utility providers trust	CIVIC_TRUST	0.644	0.639
NHS trust	HEALTH_TRUST	0.620	0.535
Academics trust	HEALTH_TRUST	0.023	0.535
Regulators trust	HEALTH_TRUST	0.020	0.598
Pharmaceutical researchers trust	HEALTH_TRUST	0.036	0.612

**Table 5:** Measurement invariance testing results for the 2022 data compared with the 2023 data in the three latent factors.

Latent Variable	Group 2023 vs. 2022 Estimate	SE	Z	*p*-Value	95% CI	Interpretation
AI_TRUST	−0.246	0.626	−0.393	0.695	[−1.472, 0.981]	No significant difference
CIVIC_TRUST	0.493	0.053	9.254	<0.001	[0.389, 0.598]	Significantly higher in 2023
HEALTH_TRUST	0.618	0.06	10.366	<0.001	[0.501, 0.735]	Significantly higher in 2023

**Table 6:** Fit indices and LPA results of the four models with AIC, BIC, entropy, and the percentage of individuals in the smallest profile for the 2022 and 2023 latent profile analysis.

		2022					2023				
Model	Classes	AIC	BIC	Entropy	Smallest n	BLRT (*p*)	AIC	BIC	Entropy	Smallest n	BLRT (*p*)
Model A (equal variances; covariances fixed to zero)	1	27,448.43	27,484.90	1.00	1.00	-	30,011.04	30,048.04	1.00	1.00	-
	2	24,512.17	24,572.95	0.77	0.35	0.01	26,878.39	26,940.06	0.77	0.37	0.01
	3	23,264.53	23,349.62	0.77	0.16	0.01	25,606.16	25,692.51	0.77	0.22	0.01
	4	22,671.98	22,781.38	0.79	0.07	0.01	24,899.05	25,010.06	0.81	0.06	0.01
	5	22,390.78	22,524.49	0.77	0.04	0.01	24,608.32	24,744.00	0.78	0.04	0.01
	6	22,309.87	22,467.90	0.74	0.03	0.01	24,433.38	24,593.73	0.76	0.04	0.01
Model B (varying variances; covariances fixed to zero)	1	27,448.43	27,484.90	1.00	1.00	-	30,011.04	30,048.04	1.00	1.00	-
	2	24,279.79	24,358.81	0.76	0.38	0.01	26,589.41	26,669.59	0.76	0.41	0.01
	3	22,951.40	23,072.97	0.76	0.23	0.01	25,175.97	25,299.32	0.79	0.19	0.01
	4	22,458.00	22,622.10	0.76	0.12	0.01	24,609.03	24,775.55	0.82	0.05	0.01
	5	22,202.32	22,408.98	0.77	0.04	0.01	24,262.79	24,472.48	0.81	0.04	0.01
	6	22,119.79	22,368.99	0.73	0.02	0.01	24,216.01	24,468.88	0.76	0.04	0.01
Model C (equal variances; equal covariances)	1	22,451.13	22,505.84	1.00	1.00	-	24,751.81	24,807.32	1.00	1.00	-
	2	22,221.30	22,300.32	0.82	0.07	0.01	24,317.35	24,397.53	0.80	0.14	0.01
	3	22,216.77	22,320.10	0.52	0.07	0.01	24,231.12	24,335.97	0.63	0.11	0.01
	4	22,062.20	22,189.84	0.59	0.04	0.01	24,162.08	24,291.59	0.64	0.06	0.01
	5	21,987.52	22,139.47	0.59	0.03	0.01	24,049.89	24,204.07	0.67	0.04	0.01
	6	21,991.30	22,167.56	0.54	0.02	0.01	24,092.08	24,270.93	0.62	0.05	0.26
Model D (varying variances; varying covariances)	1	22,451.13	22,505.84	1.00	1.00	-	24,751.81	24,807.32	1.00	1.00	-
	2	22,024.65	22,140.13	0.48	0.20	0.01	24,150.16	24,267.34	0.46	0.36	0.01
	3	21,990.05	22,166.31	0.49	0.22	0.01	24,005.88	24,184.73	0.55	0.29	0.01
	4	21,689.14	21,926.19	0.55	0.02	0.03	23,988.85	24,229.38	0.43	0.16	0.01
	5	21,964.35	22,262.18	0.45	0.13	0.01	23,889.73	24,191.93	0.51	0.13	0.01
	6	21,909.02	22,267.63	0.51	0.06	0.01	23,844.33	24,208.20	0.58	0.08	0.07

## Data Availability

The data presented in this study are available at gov.uk at https://assets.publishing.service.gov.uk/media/6361152f8fa8f50575088553/CDEI_PADAI_Tracker_Wave_2_CAWI_Weighted_data_tables.xlsx (2022 data, accessed on 2 January 2024) and https://assets.publishing.service.gov.uk/media/656f31a21104cf000dfa7559/CDEI_PADAI_Tracker_-_Wave_3_-_CAWI_-_Weighted_data_tables.xlsx (2023 data, accessed on 3 January 2024).

## Abbreviations

The following abbreviations are used in this manuscript:

AI	Artificial intelligence
AIC	Akaike Information Criterion
BIC	Bayesian Information Criterion
BLRT	Bootstrapped likelihood ratio test
CDEI	Centre for Data Ethics and Innovation
CFI	Comparative Fit Index
LPA	Latent profile analysis
MIMIC	Multiple Indicators Multiple Causes
PCA	Principal component analysis
PT	Public trust
RMSEA	Root Mean Square Error of Approximation
SEM	Structural equation model
SRMR	Standard Root Means Square Residual
UK	United Kingdom
